# The metabolomic profiling of total fat and fat distribution in a multi-cohort study of women and men

**DOI:** 10.1038/s41598-023-38318-z

**Published:** 2023-07-10

**Authors:** Rui Zheng, Karl Michaëlsson, Tove Fall, Sölve Elmståhl, Lars Lind

**Affiliations:** 1 Department of Medical Sciences, Uppsala University, Uppsala, Sweden; 2 Department of Surgical Sciences, Uppsala University, Uppsala, Sweden; 3 Division of Geriatric Medicine, Department of Clinical Sciences in Malmö, Lund University, Malmö, Sweden

**Keywords:** Biomarkers, Molecular medicine, Risk factors

## Abstract

Currently studies aiming for the comprehensive metabolomics profiling of measured total fat (%) as well as fat distribution in both sexes are lacking. In this work, bioimpedance analysis was applied to measure total fat (%) and fat distribution (trunk to leg ratio). Liquid chromatography-mass spectrometry-based untargeted metabolomics was employed to profile the metabolic signatures of total fat (%) and fat distribution in 3447 participants from three Swedish cohorts (EpiHealth, POEM and PIVUS) using a discovery-replication cross-sectional study design. Total fat (%) and fat distribution were associated with 387 and 120 metabolites in the replication cohort, respectively. Enriched metabolic pathways for both total fat (%) and fat distribution included protein synthesis, branched-chain amino acids biosynthesis and metabolism, glycerophospholipid metabolism and sphingolipid metabolism. Four metabolites were mainly related to fat distribution: glutarylcarnitine (C5-DC), 6-bromotryptophan, 1-stearoyl-2-oleoyl-GPI (18:0/18:1) and pseudouridine. Five metabolites showed different associations with fat distribution in men and women: quinolinate, (12Z)-9,10-dihydroxyoctadec-12-enoate (9,10-DiHOME), two sphingomyelins and metabolonic lactone sulfate. To conclude, total fat (%) and fat distribution were associated with a large number of metabolites, but only a few were exclusively associated with fat distribution and of those metabolites some were associated with sex*fat distribution. Whether these metabolites mediate the undesirable effects of obesity on health outcomes remains to be further investigated.

## Introduction

Obesity, characterized by excessive body fat mass, is a burden to the public health, due to its causal role in both late-onset diseases and early mortality^1^. Lifestyle changes have driven obesity prevalence to epidemic proportions, both in industrialized and developing countries^2,3^. Central obesity (abdominal fat accumulation) has been suggested as especially detrimental for health and a more sensitive indicator of metabolic dysregulations^4^, and a better predictor of cardiovascular events than the general obesity^5^. Sex-difference in body shape and fat distribution is well known, which leads to different tendency of abdominal fat accumulation and associations with cardiometabolic risk factors in men and women^6–9^.

Untargeted metabolomics can profile thousands of metabolites simultaneously in a sample and has been widely implemented to explore biochemical signatures of specific phenotypes in the last decade^10–12^. Investigating central obesity-associated metabolites could provide us with a better understanding of the etiology of related diseases, potentially facilitating the discovery of actionable targets for prevention.

To date, a few metabolomics studies of obesity (mostly represented by BMI or waist circumference) have been reported^13–19^, but studies using both measurements of fat mass and an untargeted approach are lacking. Furthermore, to our knowledge there is no direct comparison of the metabolic signatures of total body fat and fat distribution reported, let alone discovering whether such metabolic signatures differ by sex.

Large-scale estimation of body composition by use of bioimpedance has advantages such as ease of use, non-invasiveness, non-radioactive, and low cost^20^. The technical improvement of segmental multifrequency system, allows bioimpedance analysis (BIA) to evaluate regional body composition^21,22^. In this study, our primary aim was to identify metabolites associated with total body fat (%) and fat distribution measured by BIA, using information from participants in three Swedish cohorts in a discovery-replication fashion. A secondary aim was to identify metabolites differentially associated with either of the two fat mass measures in men and women.

## Materials and methods

### Study cohorts

The Epidemiology for Health (EpiHealth) study was initiated in 2011^23^. A random sample of individuals aged 45–75 who were living in Uppsala and Malmö, Sweden, were invited to a health screening survey by ordinary mail. Participants were asked to undergo a physical examination at the clinical center. In 2018, in total 25,350 subjects were included. Plasma samples from a random subgroup of 2342 subjects (50% women) recruited in Uppsala were collected and further used for metabolomics analysis. The minimal fasting period in this cohort was 6 h.

The Prospective investigation of Obesity, Energy and Metabolism (POEM) study was conducted in Uppsala between 2010 and 2016. A total of 502 participants (50% women) aged 50 were included, and all participants provided plasma samples for metabolomics analysis after an overnight fast^24,25^.

Between 2001 and 2004, a random sample of residents aged 70, in Uppsala were invited to participate the Prospective Investigation of the Vasculature in Uppsala Seniors (PIVUS) study. In total, 1016 participants underwent a health examination. Two additional examinations were conducted at the ages of 75 and 80 years and overnight-fasting blood samples were obtained. The current study used metabolomics data from 603 participants from the 80-year examination (50% women)^24^.

All blood samples were drawn between 8 and 10 am at the same day as the bioimpedance measurements. The characteristics of the three cohorts are shown in Table 1. The three studies were approved by the Uppsala University ethics committee (Application numbers 2010/402, 2011/045 and 2009/057), performed in accordance with the Declaration of Helsinki and other relevant guidelines/regulations, and informed written consent was obtained from all participants.

**Table 1:** Basic characteristics of the three cohorts.

	EpiHealth	POEM	PIVUS
n	2342	502	604
Age (years)	61 (8.4)	50 (0.1)	80 (0.2)
Female sex (%)	50%	50%	50%
BMI (kg/m^2^)	26.5 (3.8)	26.4 (4.2)	26.9 (4.6)
Waist/hip ratio (WHR)	0.90 (0.08)	0.90 (0.08)	0.90 (0.07)
Total fat (%)			
All	30 (8.0)	28 (8.0)	32 (8.0)
Men	24 (5.2)	22 (5.2)	27 (6.2)
Women	36 (6.5)	33 (7.2)	37 (7.5)
Fat distribution (trunk/leg fat mass ratio)			
All	1.04 (0.26)	1.00 (0.25)	1.04 (0.28)
Men	1.25 (0.20)	1.20 (0.17)	1.27 (0.20)
Women	0.82 (0.11)	0.81 (0.15)	0.81 (0.12)
Alcohol intake	2.43 (2.92) (drinks/week)	NA	NA
Exercise habits	2.29 (0.80) (On a 5-grade scale)	2.80 (1.01) (On a 4-grade scale)	1.21 (1.31) (On a 4-grade scale)
Education (%)			
< 10 years	21	8	56
10–12 years	29	44	19
> 12 years	50	48	25

Means and standard deviations (in parenthesis) or proportions are given.

### Covariates from questionnaire data

Life-style factors were evaluated using a questionnaire in all samples. In EpiHealth, leisure-time physical activity was assessed on a 5-level scale with 1 as sedentary and 5 as athlete training. Smoking was defined as the sum of years smoking. Alcohol intake was assessed as drinks per week. In the POEM and the PIVUS cohorts, leisure-time physical activity was assessed on a 4-level scale with 1 as sedentary and 4 as athlete training. Smoking variable was classified as current smoking vs non-smoking. Alcohol intake was not assessed in the PIVUS cohort at age 80 years, and not all in the POEM cohort. In all three cohort studies, education was defined on a three-level scale: < 10, 10–12 and > 12 years in school.

The degree of missingness on covariates was low and list-wise deletion was used.

### Fat mass measurement

In all three cohorts, fat mass and body weight were assessed through a weight scale that also calculates total fat mass by mean of bioimpedance (Tanita BC-418MA, Tokyo, Japan). This equipment also calculates fat mass separately in the arms, trunk region and the legs using electrodes connected both to the feet and the hands. In the present study, we denoted the total fat mass divided by body weight as “total fat (%)”, while the ratio of trunk to leg fat mass was denoted as “fat distribution”.

In the POEM study, total and regional fat mass were additionally measured by whole body scans by DXA (Lunar Prodigy, GE Healthcare)^26,27^. The validity of fat mass derived by Lunar Prodigy has been evaluated against the 4-compartment model, the tool that is currently considered the gold standard method of body composition appraisal, resulting in 1.7–2.0% higher fat mass estimates with this narrow fan-beam DXA equipment^28^. We assessed the concordance of the bioimpedance method against DXA, using linear regression to obtain the correlation coefficients and also generated Bland–Altman plots of total body and regional fat mass measures. Only POEM data were used for this analysis.

### LC–MS metabolomics

Untargeted metabolomics was conducted on plasma samples from all the three studies applying the Metabolon platform (Metabolon Inc., USA). The methods have been described in detail elsewhere^29^. In brief, samples were prepared automatically using the MicroLab STAR® system of Hamilton Company. Quality control was conducted by using pooled analytical samples, blank and chemical standards as detailed previously^30^. Proteins were precipitated with methanol under vigorous shaking for 2 min (Glen Mills GenoGrinder 2000) followed by centrifugation. The resulting extract was then divided into five fractions: two for analysis by two separate reversed-phase (RP)/UPLC-MS/MS methods with electrospray ionization (ESI) positive mode, one for analysis by RP/UPLC-MS/MS with ESI negative mode, one for analysis by hydrophilic interaction (HILIC)/UPLC-MS/MS with ESI negative mode, and one sample was reserved for backup. In the current analyses, only identified, non-xenobiotic metabolites with a detection rate over 75% in all samples were included (n = 791). To correct batch effect, the raw values of each metabolite in the experimental samples were divided by the median of those samples in each instrument batch. For each metabolite, the minimum value across all batches was imputated for missing values. Then the values were imputed and presented in an arbitrary unit. Metabolite identification was achieved by comparison to library entries of purified standards based on molecular weight, retention time, preferred adducts, in-source fragments and associated MS spectra^29^.

### Statistics

To test potentially non-linear relationship between total fat (%) and fat distribution, we performed a linear regression model including a restricted cubic spline function for total fat (%) with three knots (10th, 50th and 90th decentiles). This analysis was only conducted in the EpiHealth sample.

Four linear regression models were performed for each metabolite. The metabolite was used as the dependent variable. In the first set of models, total fat (%) or fat distribution (ln-transformed) was the independent variables evaluated with age, sex, date of visit, education, exercise habits, smoking and alcohol intake as covariates. In the second set of models, an interaction term between total fat (%) or fat distribution and sex was included. In the third step, the first model was run in men and women separately.

All of these models were run in the three cohorts separately. The results from the EpiHealth study were used as the discovery step and the metabolites with a Benjamini–Hochberg false discovery rate (FDR) < 0.05 were brought forward to the replication step. The replication step was a meta-analysis of the results from the POEM and PIVUS studies, meta-analyzed with inverse-variance weighted (IVW) fixed effect models. Also in the validation step, we considered FDR < 0.05 and consistent effect direction for a significant validated finding. The same discovery/replication procedure was employed also for the sex-interaction evaluation and the sex-stratified analyses. STATA16.1 was used for analysis (Stata Inc, College Station, TX, USA).

The metabolite findings were subjected to a targeted pathway enrichment and topology analysis using MetaboAnalyst 5.0 (https://www.metaboanalyst.ca/MetaboAnalyst/home.xhtml), which is an online repository for metabolomics analysis and interpretation, that applies the Kyoto Encyclopedia of Genes and Genomes (KEGG) compound database to perform overrepresentation pathway enrichment analysis. Only annotated metabolites having a human metabolome database (HMDB) identifier were included. The hypergeometric test was used for the enrichment analyses.

## Results

**Figure 1:**
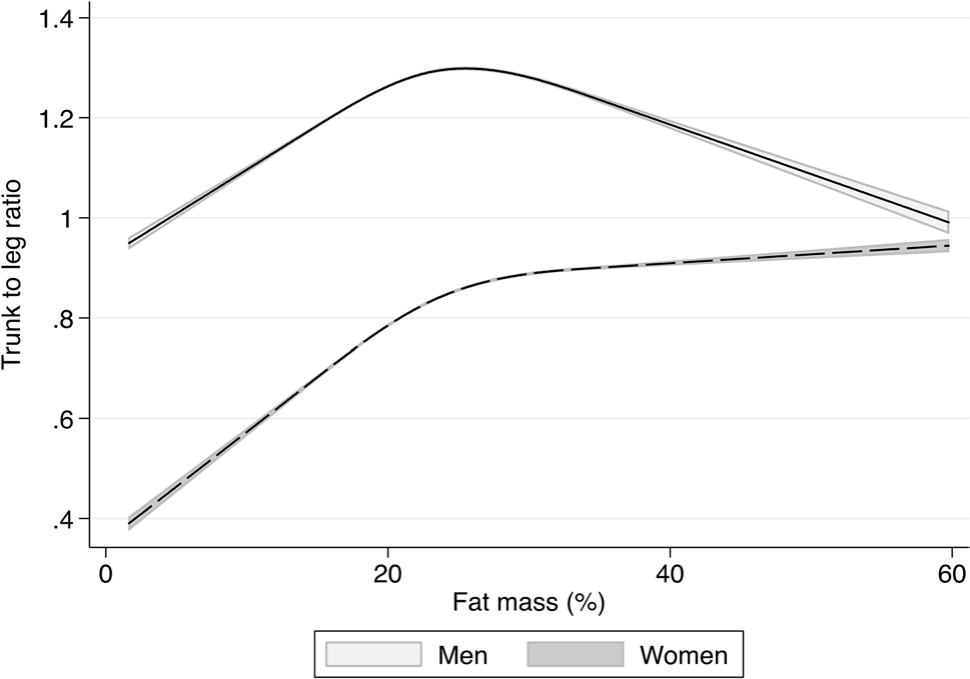
Relationship between total fat (%) and fat distribution (trunk to leg ratio) in the EpiHealth study. A spline function with three knots was used (10th, 50th and 90th percentile).

Basic characteristics of the three cohorts are given in Table 1. As shown in Fig. 1, a non-linear and nonmonotonous association between total fat (%) and fat distribution could be observed, which also differed by sex. Such an observation further motivated us to profile the metabolic signatures of total fat (%) and fat distribution separately and to investigate their interactions with sex.

### Total fat (%) associated metabolites

**Figure 2:**
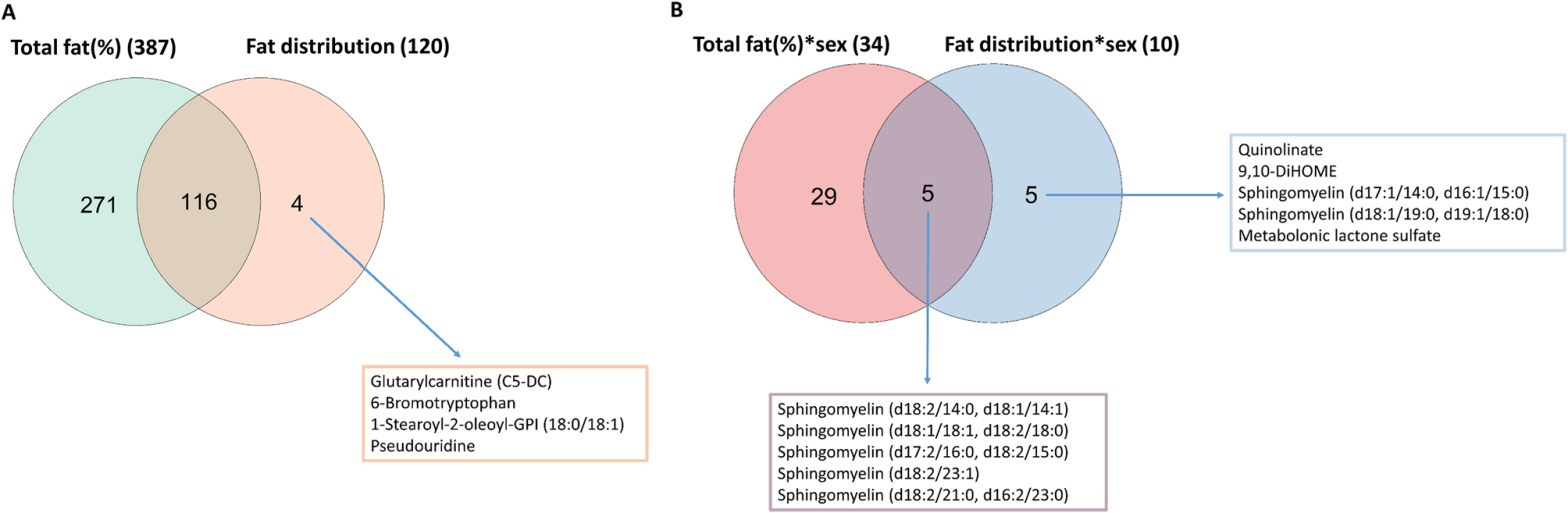
Venn diagram for replicated metabolites associated with total fat (%) or fat distribution (**A**) or their interactions with sex (**B**).

In the discovery phase, total fat (%) was associated with 539 of the 791 evaluated metabolites after the adjustment for age, sex and life-style factors. Of those, 387 were replicated in the validation phase (Supplementary Table S1, Fig. 2A). The largest effect sizes were noted for total fat (%) when associated with cortolone glucuronide (β = 0.064, *p* < 0.001, R^2^ = 0.22), metabolonic lactone sulfate (β = 0.059, *p* < 0.01, R^2^ = 0.25), 1-linoleoyl-GPC (18:2) (β =  − 0.058, *p* < 0.001, R^2^ = 0.22), sphingomyelin (d18:0/18:0, d19:0/17:0) (β = 0.050, *p* < 0.001, R^2^ = 0.17), and N-stearoyl-sphinganine (d18:0/18:0) (β = 0.048, *p* < 0.001, R^2^ = 0.16). Table 2 shows the pathway enrichment analysis for the 387 replicated metabolites. Aminoacyl-tRNA biosynthesis, amino acid synthesis and metabolism were the top enriched pathways.

**Table 2:** Pathway enrichment analysis for the 387 replicated metabolites associated with total fat (%).

Metabolite set	Hits	Expected	Total	*P*-value
Aminoacyl-tRNA biosynthesis	15	2.91	48	3.84E−08
Valine, leucine and isoleucine biosynthesis	6	0.49	8	1.08E−06
Arginine biosynthesis	7	0.85	14	5.86E−06
Glycine, serine and threonine metabolism	10	2.00	33	1.21E−05
Alanine, aspartate and glutamate metabolism	9	1.70	28	2.01E−05
Cysteine and methionine metabolism	9	2.0013	33	8.69E−05
Pantothenate and CoA biosynthesis	6	1.15	19	6.05E−04
Phenylalanine, tyrosine and tryptophan biosynthesis	3	0.24	4	8.27E−04
Glycerophospholipid metabolism	8	2.18	36	1.00E−03
Sphingolipid metabolism	6	1.27	21	1.10E−03
D-Glutamine and D-glutamate metabolism	3	0.36	6	3.78E−03
Taurine and hypotaurine metabolism	3	0.49	8	9.68E−03
Nicotinate and nicotinamide metabolism	4	0.91	15	1.03E−02
Glyoxylate and dicarboxylate metabolism	6	1.94	32	1.06E−02
Histidine metabolism	4	0.97	16	1.31E−02
Phenylalanine metabolism	3	0.61	10	1.90E−02
Nitrogen metabolism	2	0.36	6	4.65E−02

Total, total number of metabolites in this metabolite set (pathway); expected, expected number of metabolites projected to each set; hits, observed number of metabolites of interest projected to each set.

**Figure 3:**
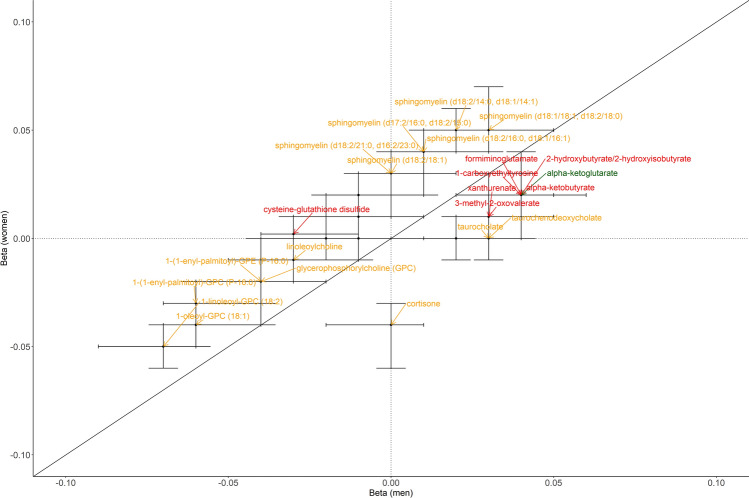
Betas for total fat (%) in men and women in the associations with the 34 metabolites with a validated sex-total fat (%) interaction. Highlighted metabolites were labelled red, green or orange if they are amino acids, energy metabolite or lipids, respectively.

In the discovery phase, a significant interaction between sex and total fat (%) was found for the association of total fat (%) with 115 metabolites. Of those, the interaction effect was replicated for 34 metabolites (Fig. 2B), belonging to amino acids, energy metabolites and lipids. As seen in Fig. 3, the betas were higher in women than men for 23 out of these 34 metabolites and most of these metabolites are lipids. In contrast, the associations of total fat (%) with most amino acids were stronger in men than women. Total fat (%) had negative associations with lysophospholipids, including 1-oleoyl-GPC (18:1) and 1-linoleoyl-GPC (18:2) whereas had positive associations with sphingomyelins, such as sphingomyelin (d18:1/18:1, d18:2/18:0) in both women and men. Total fat (%) were associated with two bile acids, taurocholate and taurochenodeoxycholate only in men (β = 0.03, *p* < 0.001, R^2^ = 0.03) and were associated with cortisone only in women (β =  − 0.04, *p* < 0.001, R^2^ = 0.05). The detailed statistics of these total fat (%)-34 metabolites associations in men and women are given in Supplementary Table S2.

### Fat distribution-associated metabolites

In the discovery phase, fat distribution was associated with 166 of the 791 evaluated metabolites after adjustment for age, sex and life-style factors. Of those, 120 were replicated in the validation phase (Supplementary Table S3, Fig. 2A). The largest effect sizes were noted for cortolone glucuronide (β = 1.38, *p* = 1.86e−14, R^2^ = 0.10), 1-linoleoyl-GPC (18:2) (β =  − 1.16, *p* = 6.46e−11, R^2^ = 0.11)), 1-(1-enyl-palmitoyl)-2-linoleoyl-GPC (P-16:0/18:2) (β =  − 1.14, *p* = 1.46e−10, R^2^ = 0.12), gamma-glutamylvaline (β = 1.10, *p* = 6.32e−11, R^2^ = 0.11) and gamma-glutamylphenylalanine (β = 1.10, *p* = 1.98e−10, R^2^ = 0.12). Total fat (%) and fat distribution shared a great number of associated metabolites and only four metabolites linked with fat distribution only, including glutarylcarnitine (C5-DC), 6-bromotryptophan, 1-stearoyl-2-oleoyl-GPI (18:0/18:1) and pseudouridine. A pathway enrichment analysis of the replicated metabolites disclosed aminoacyl-tRNA biosynthesis, amino acid synthesis and metabolism, glycerophospholipid metabolism and sphingolipid metabolism to be amongst the enriched pathways (see Table 3 for details).

**Table 3:** Pathway enrichment analysis for the 120 replicated metabolites associated with fat distribution.

Metabolite set	Hits	Expected	Total	*P*-value
Aminoacyl-tRNA biosynthesis	7	0.96	48	2.61E−05
Valine, leucine and isoleucine biosynthesis	3	0.16	8	3.80E−04
Glycerophospholipid metabolism	5	0.72	36	5.58E−04
Histidine metabolism	3	0.32	16	3.40E−03
Sphingolipid metabolism	3	0.42	21	7.55E−03
Alanine, aspartate and glutamate metabolism	3	0.56	28	1.69E−02
Cysteine and methionine metabolism	3	0.66	33	2.63E−02
Arginine biosynthesis	2	0.28	14	3.03E−02
Nicotinate and nicotinamide metabolism	2	0.30	15	3.46E−02
Glycerolipid metabolism	2	0.32	16	3.90E−02
Valine, leucine and isoleucine degradation	3	0.80	40	4.34E−02

Total, total number of metabolites in this metabolite set (pathway); expected, expected number of metabolites projected to each set; hits, observed number of metabolites of interest projected to each set.

**Figure 4:**
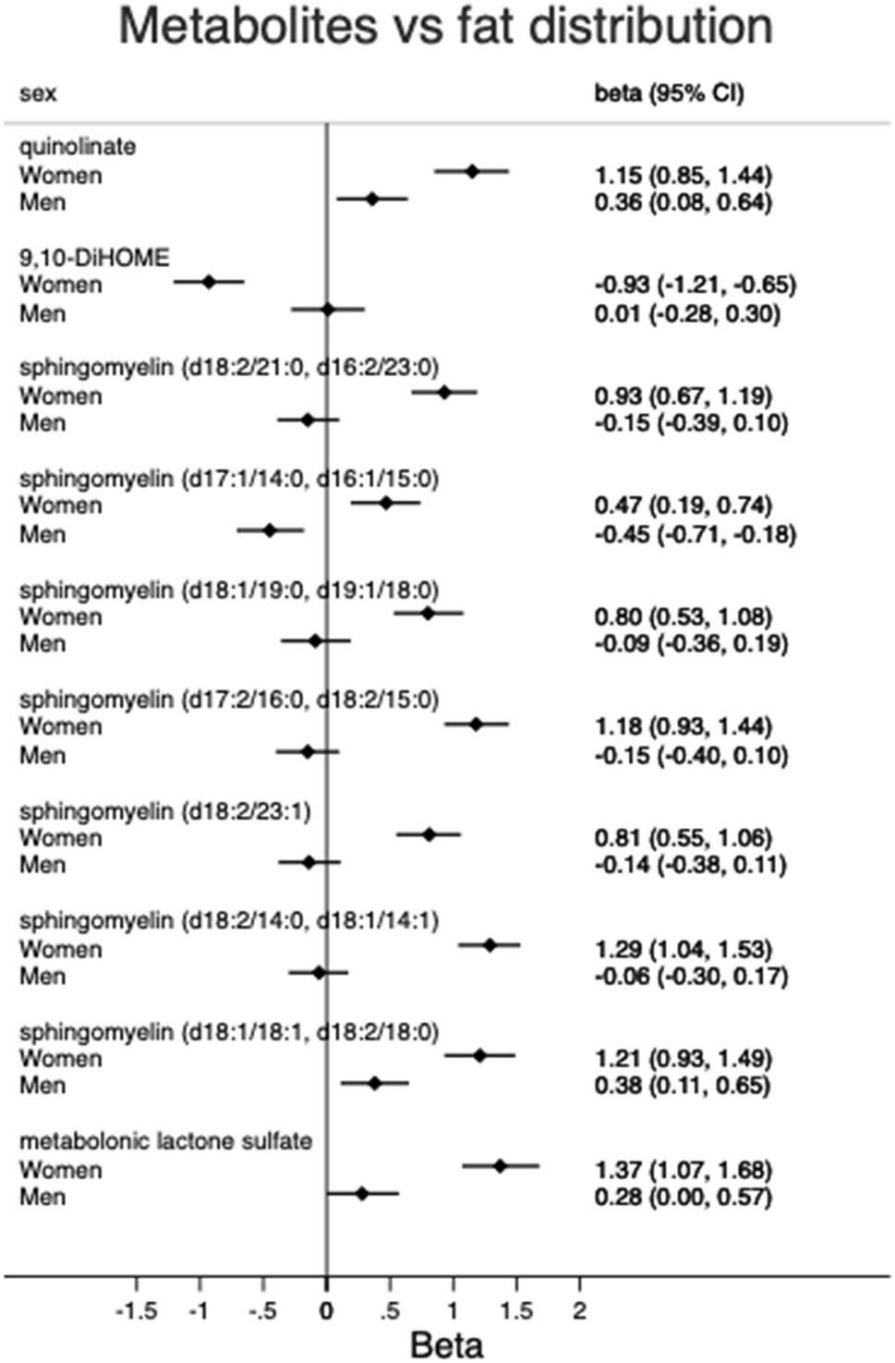
Estimates for fat distribution (trunk to leg ratio) in the associations with 10 metabolites with a validated sex-fat distribution interaction in men and women.

In the discovery phase, a significant interaction between sex and fat distribution was found for the association of fat distribution with 43 metabolites. Of those, 10 were replicated. As seen in Fig. 4, the relationships of fat distribution with all ten metabolites were stronger in women and most of the replicated metabolites are sphingomyelins which positively associated with more fat distributed to trunk in women. Except sphingomyelin (d18:1/18:1, d18:2/18:0), the rest of sphingomyelins tended to negatively associate with fat distributed to trunk in men but only sphingomyelin (d17:1/14:0, d16:1/15:0) had a statistically significant estimate. Five sphingomyelins of the ten metabolites also associated with the interaction of total fat (%) and sex, and the rest associated with fat distribution-sex interaction only (Fig. 2B), including quinolinate, 9,10-DiHOME, sphingomyelin (d17:1/14:0, d16:1/15:0), sphingomyelin (d18:1/19:0, d19:1/18:0) and metabolonic lactone sulfate. In women, 9,10-DiHOME was the only metabolite negatively that associated with fat distribution and metabolonic lactone sulphate was the most pronounced fat distribution-associated metabolite.

### BIA versus DXA-measured total fat (%)

As shown in Supplementary Figure S1 A, the measurements of total fat (%) by bioimpedance and DXA were highly correlated (r = 0.93, *p* < 0.0001), but the bioimpedance technique tended to underestimate the total fat (%) compared to DXA. Similarly, at the three different locations, arm, leg and trunk, high correlations were seen between the two techniques (r = 0.93 for arm, r = 0.90 for leg and r = 0.87 for trunk, *p* < 0.0001 for all, Supplementary Figure S1 C, E and G). An underestimation of trunk fat (%) by BIA was also seen compared to DXA. As could be seen in the Bland–Altman plots (Supplementary Figure S1 B, D, F and H), the underestimation of total fat (%) by bioimpedance was most exaggerated at high levels. The same pattern was seen for trunk fat.

## Discussion

In this study, we discovered and replicated the associations of total fat (%) or fat distribution with a large number of plasma metabolites. Some of these metabolites were mainly related to the fat distribution. Also metabolites showing different associations in men and women with fat distribution were highlighted.

A previous study showed a positive, but very weak, correlation between general obesity (measured by body mass index) and trunk-to-leg fat mass ratio^31^. However, the relationship between general and central obesity is more complicated as shown in our work and cannot be simply captured by a correlation coefficient.

Metabolites mainly related to fat distribution were glutarylcarnitine (C5-DC), 6-bromotryptophan, 1-stearoyl-2-oleoyl-GPI (18:0/18:1) and pseudouridine. C5-DC is a catabolic product of branched-chain amino acid and has been associated with insulin resistance and increased cardiovascular events^32,33^. 6-Bromotryptophan, which might be produced by the gut microbiota^34^, has been associated with chronic kidney disease progression^35^, but a relationship to fat distribution has not been reported previously. Studies highlighting1-stearoyl-2-oleoyl-GPI (18:0/18:1) and its associations with obesity are elusive. Pseudouridine is involved in pyrimidine metabolism and has been found to be strongly perturbed by obesity and is also associated with type 2 diabetes^36,37^.

Cortolone glucuronide was the top metabolite linked with both total fat (%) and fat distribution, and this finding is in agreement with a previous Canadian study using BMI as the measure of obesity^38^. Cortolone glucuronide is a steroidal metabolite and found increased in individuals with metabolic syndromes and gestational diabetes mellitus^39,40^. Metabolonic lactone sulphate was linked with both total fat (%) and fat distribution, while different effects by sex was found only for fat distribution. Metabolonic lactone sulphate is a steroid-like metabolite which has been linked to the circulating levels of several androgens^41^. Previous studies have found positive associations of this metabolite with BMI^42^, liver fat percentage and visceral adipose tissue volume^29^, as well as several other cardiometabolic risk factors^43–45^.

The enrichment analysis suggested that several metabolic pathways are shared by general and central obesity, including aminoacyl-tRNA biosynthesis, several amino acids biosynthesis and metabolism, glycerophospholipid metabolism, sphingolipid metabolism and nicotinate and nicotinamide metabolism. Given that a much larger number of metabolites were associated with general obesity, additional enriched pathways were found for total fat (%), such as glycine, serine and threonine metabolism as well as pantothenate and CoA biosynthesis. It is striking, in this analysis, that the aminoacyl-tRNA biosynthesis outranked the lipid metabolism pathways given the close relation of lipids with adipose tissue expansion^46,47^. Aminoacyl-tRNA biosynthesis reflects the use of amino acids in the synthesis of proteins^23^. Indeed, an alteration of protein synthesis rate in obese individuals has been observed previously^48,49^, which might be caused by the obesity-induced lipotoxicity to the incorporation of amino acid in the skeletal muscle proteins^49^. A proteomics study further implied that such an adverse effect is similar in abdominal and femoral adipose tissues^50^.

The biosynthesis of branched-chain amino acids (BCAAs) ranked the second most significantly enriched pathway. The close but complex relationships between BCAAs and obesity have been observed over decades^32,51,52^. Elevated levels of BCAAs can be observed when BCAA metabolism is impaired in insulin-resistant obese individuals^53^. In addition, more recent evidence has suggested that BCAA is a substantial resource of lipogenesis via mitochondrial BCAA catabolism, which is suppressed in obese adipose tissue by hypoxia^54^.

Both of lysoglycerophospholipids and sphingomyelins were linked with general obesity, but the associations with these two lipid species were in different directions, as reported previously^55^. Those relationships were generally concordant between men and women albeit more pronounced in women. The current understanding of the role of lysoglycerophospholipids and sphingomyelins in obesity is limited to those located in the tissue and cellular membrane^55,56^, but the interplay between stored and circulating levels is elusive and thus hard to comprehend. Sphingomyelins were the dominant metabolite species associated with central obesity where sexual dimorphism can be observed. The mechanism behind this sex-discrepancy is not yet fully understood, but the difference in insulin response and estrogen metabolism between women and men might play a role^57,58^. Notably, the length and saturation of the fatty acyl chain seem to make a difference in the associations of sphingomyelins with central obesity as the current result suggested, which agrees with a previous study^59^.

Several factors are known to influence the metabolome, such as food intake, season, fasting time, time of the day for blood sampling, the microbiome, etc. In the present study, we have tried to control for some of these factors by drawing the blood after at least 6 h of fasting at the same time of the day (8–10 am) and including the date as a covariate. Some factors were not controlled for, such as diet intake or the microbiome, since those data were not available in all cohorts. To further reduce the influence of unmeasured factors, we performed a discovery/validation evaluation, so the reported findings were seen in all three different cohorts making chance findings in one sample only not be reported as significant. Thus, even though not all factors affecting the metabolome were controlled for, the current design was conservative and not likely to produce false positive findings.

The strength of this study is that we used a discovery-replication study design in three population-based cohorts with great coverage of metabolites. Additionally, we measured total and regional fat (%) instead of using anthropometrics, which more likely reflects the fat accumulation and distribution of the body in a more accurate fashion. In addition, we validated this bioimpedance method vs the DXA technique and found a good agreement.

One limitation is that the current analysis was cross-sectional and no causality or casual direction can be inferred. Because of the large diversity and number of metabolites, curation of genetic instruments for specific metabolites to be subject to Mendelian randomization remains challenging. In addition, horizontal pleiotropy might be a major problem provided that many obesity-associated metabolites are influenced by the same sets of genetic variants and also closely connected in metabolic pathways.

It should be acknowledged that the fasting time at blood collection was shorter (although 6 h) in EpiHealth compared to the other two samples. However, even if this might affect some metabolites, this discrepancy could only drive the associations in the discovery/validation framework towards the null hypothesis and will not produce any false positive findings. Furthermore, the current findings are based on individuals of European ancestry and to generalize these findings to other populations, validation studies are warranted in the future.

To conclude, total fat (%) and fat distribution were robustly associated with many metabolites. A great overlap of metabolites linked with the two phenotypes was found, featuring several steroid-like metabolites, lysophospholipids, sphingomyelins and branched-chain amino acids, but some associations mainly for fat distribution could be observed. Some of these associations also differed by sex. Our results could provide motivations to further evaluate if metabolites mainly associated with fat distribution could be mediators of the deleterious effect of central obesity on multiple health outcomes, such as diabetes and cardiovascular diseases.

## Supplementary Information

Supplementary Information.

Supplementary Tables.

## Data Availability

Due to Swedish laws on personal integrity and health data, as well as the decision by the Ethics Committee, we are not allowed to make any data including health variables open to the public, even if made anonymous. The data could be shared with other researchers after a request to the steering committee (karl.michaelsson@surgsci.uu.se).
