# Supplementary material for: The metabolomic profiling of total fat and fat distribution in a multi-cohort study of women and men
Source: Sci Rep. 2023 Jul 10;13:11129. doi: 10.1038/s41598-023-38318-z (PMC10333321; doi:10.1038/s41598-023-38318-z)
Supplement: Supplementary file 1 — Supplementary Information. [file 41598_2023_38318_MOESM1_ESM.docx]

**Supplementary materials for:**

**The metabolomic profiling of total fat and fat distribution**

**in a multi-cohort study of women and men**

**Rui Zheng^1*^, Karl Michaëlsson^2^, Tove Fall^1^, Sölve Elmståhl^3^ and Lars Lind^1^**

1. **Department of Medical Sciences, Uppsala University, Uppsala, Sweden**
2. **Department of Surgical Sciences, Uppsala University, Uppsala, Sweden**
3. **Division of Geriatric Medicine, Department of Clinical Sciences in Malmö, Lund University, Malmö, Sweden**

***Corresponding: rui.zheng@uu.se**

**Supplementary method:**

**Model 1:**

***All samples for each of the three cohort***

**Metabolites ~ total fat (or fat distribution) + age + sex+ date of visit + education + exercise habits+ smoking + alcohol intake**

**Model 2:**

***All samples for each of the three cohort***

**Metabolites ~ total fat (or fat distribution) + age + sex+ date of visit + education + exercise habits+ smoking + alcohol intake + sex*** **total fat (or fat distribution)**

**Model 3:**

***Models were run for women and men separately for each of the three cohorts***

**Metabolites ~ total fat (or fat distribution) + age + date of visit + education + exercise habits+ smoking + alcohol intake**


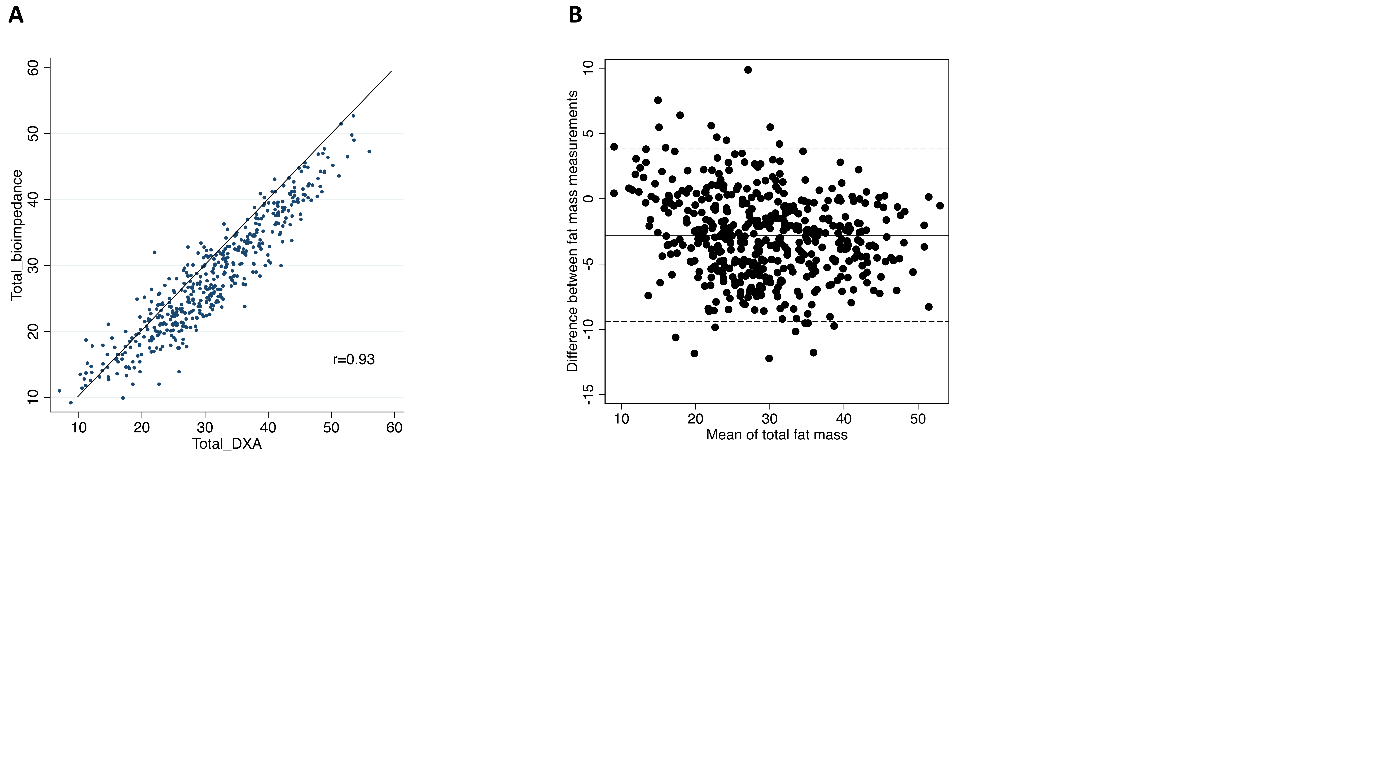


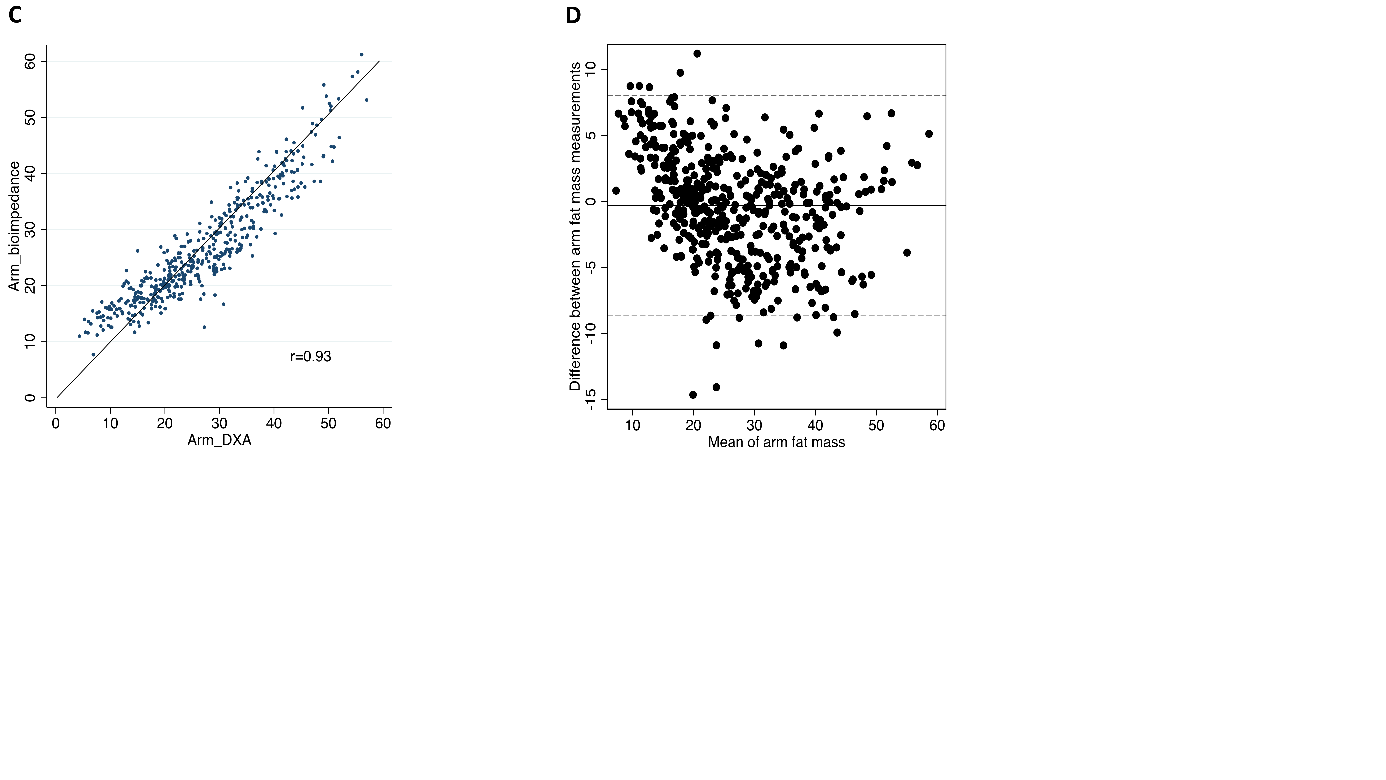


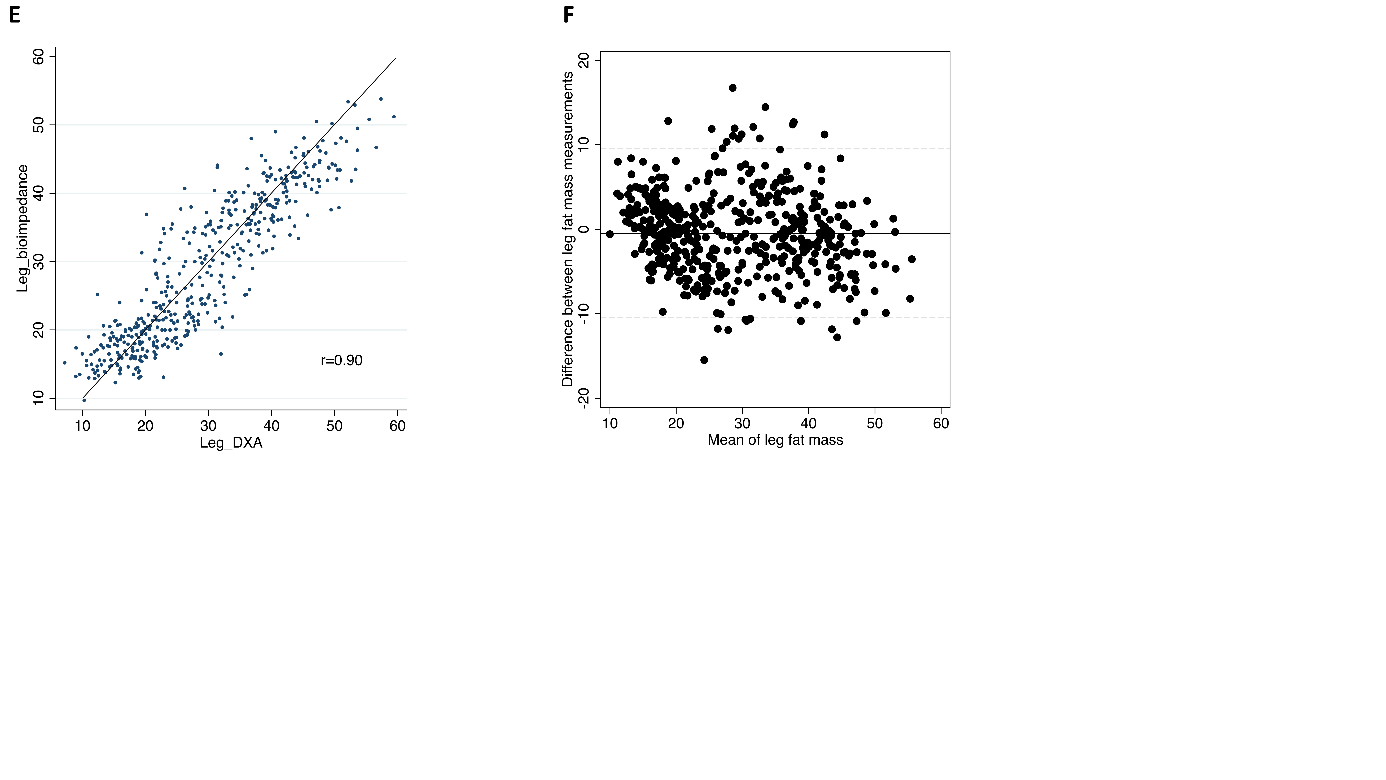


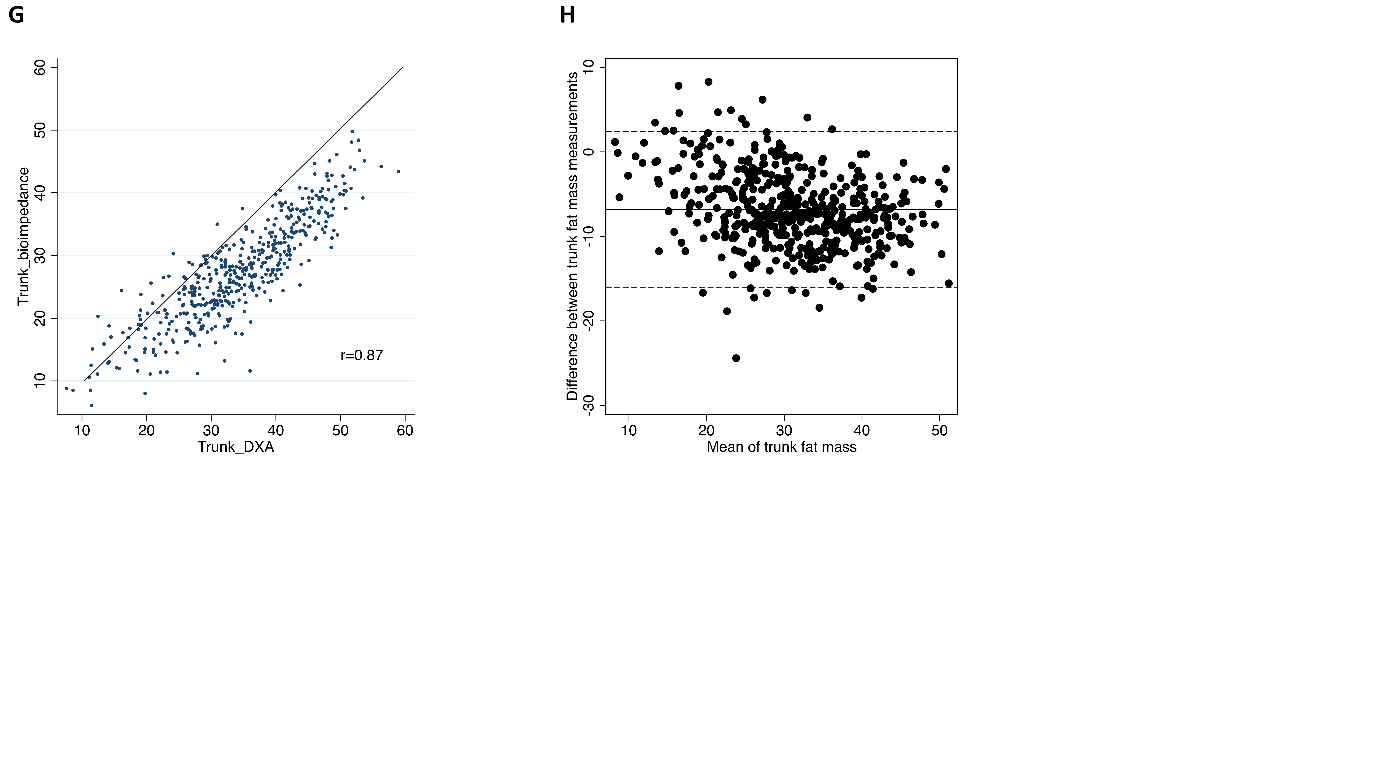


Figure S1. Relationship between total and regional fat (%) measured by bioimpedance and DXA. Pearson correlation plot is shown on the left panel and the line of identity is given. On the right panel, Bland-Altman plot shows the mean of the two methods vs the difference (bioimpedance – DXA). Solid and dashed lines indicate the mean difference and two standard deviations of the difference.
